# Chemical Composition and In Vitro Evaluation of the Mosquito (Anopheles) Repellent Property of Neem (*Azadirachta indica*) Seed Oil

**DOI:** 10.1155/2021/5567063

**Published:** 2021-06-04

**Authors:** Olive Aidoo, Noble Kuntworbe, Fredrick William Akuffo Owusu, Deryl Nii Okantey Kuevi

**Affiliations:** Department of Pharmaceutics, Kwame Nkrumah University of Science and Technology, Kumasi, Ghana

## Abstract

As one of the killer diseases in the world, malaria contributes to child mortality and global death annually. As a result, many reactive mechanisms have evolved to control and repel mosquitoes. The use of synthetic mosquito repellents with N,N-Diethyl-meta-toluamide (DEET) is one of the popular interventions despite its dermatological limitations such as skin irritations. Ethnobotanical reviews have identified that the adoption of natural repellents promises high repellence on mosquitoes with minimal side effects compared with synthetic ones. However, this has received little attention in modern pharmaceutical literature. This research is focused on the formulation of a natural mosquito repellent from the oil extracted from *Azadirachta indica* (A. Juss). The oil cream was formulated in concentrations of 10% *v*/*w*, 12.5% *v*/*w*, 15% *v*/*w*, 17.5% *v*/*w*, and 20% *v*/*w* using an in vitro evaluation approach. Pharmacopoeia characteristics of the cream such as pH, viscosity, spreadability, and organoleptic properties were carried out to verify acidity, permeation, and flow properties of the formulated cream. The spreadability rate was inversely proportional to the concentration of the cream in terms of oil content falling from 1.24 gm/s to 0.84 gm/s from concentrations 10% *v*/*w* to 20% *v*/*w* correspondingly. Skin irritation tests, however, indicated traces of irritation at 20% *v*/*w*. Repellency properties of the cream revealed a lasting effect on the mosquitoes, although this was dependent on concentration levels. Formulations of 17.5% *v*/*w* and 20% *v*/*w* neem seed oil cream had an equal repellency effect of 87.5%, whereas the synthetic repellent had a repellency of 75% within a justifiable time frame for all the formulations. This work shows that plant-based mosquito repellents promise a healthier approach in controlling mosquito bites, protecting the skin, and preventing malaria.

## 1. Background

The mosquito parasite is notoriously known in the public health domain as a causative transmitter of various diseases such as dengue fever, chikungunya, filariasis, and malaria. It is prevalent in humid and sub-tropical regions including most parts of sub-Saharan Africa, America, and Asia. Transmission of malaria occurs through the bites of the female anopheles mosquito. Malaria has received tremendous attention over the years and is still considered one of the killer diseases of the century. For instance, it was estimated by WHO in 2018 that about 219 million people contracted malaria, and out of this number, 435,000 died. In 2019, infections increased to 229 million with 409,000 deaths with over 60% of these deaths being predominantly children. Thus, the prevention and elimination of malaria should receive continuous attention and research. Currently, vector control methods such as indoor residual spraying (IRS), sleeping in insecticide-treated nets (ITNs), and the use of mosquito repellents are the most effective ways of breaking the cycle of human to mosquito to human transmission of malaria [1]. The use of mosquito repellents is particularly important in preventing mosquito bites and hence transmission of malaria [2]. The mosquito repellents presently available for use by patients on the Ghanaian market have N,N-Diethyl-meta-toluamide (DEET) as the active ingredient. DEET is very effective in preventing mosquito bites; however, there have been accounts of toxicity complications such as contact urticaria and skin rashes [3]. Furthermore, access to efficient repellent products remains a challenge in some communities in Ghana. In poor communities in Ghana, people have adopted conventional means of repelling mosquitoes [4]. For example, in some parts of Ghana, oil expressed from neem seed is applied directly to the skin to prevent mosquito bites. Again, scientific studies have proven that concentrations of neem oil above 7.5% have a relatively higher potential of repelling mosquitoes compared with DEET [5]. Various types of repellents exist in different topical formulations on the Ghanaian market. This includes creams, ointments, lotions, gels, and pastes. Creams are pharmaceutical products prescribed for the treatment of external disorders. The term is also used to describe disperse systems in which one insoluble phase is dispersed as droplets within a second liquid phase [6]. They are pseudoplastic systems with suitable consistency; however, they are fundamentally unstable that in the absence of emulsifying agents will separate them into two separate phases [7].

According to Geetha and Anitha [8], creams are emulsions of water and oil. Principally, they are classified as oil in water (*o*/*w*) or water in oil (*w*/*o*) emulsions. In the former systems, the oil (or internal) phase is dispersed as droplets through the external aqueous phase. Conversely, in *w*/*o* creams, the internal phase is composed of water droplets, and the external phase is nonaqueous. Moreover, *o*/*w* creams spread easily and do not leave the skin greasy and sticky. Additionally, *w*/*o* creams are greasier and more emollient. Moreover, since this type of cream restricts evaporation from the skin, it can be used on nonweeping surfaces to prevent dehydration. The flow properties of the cream should enable the formulation to be easily removed from the container. Furthermore, the formulation must spread over the affected area and be aesthetically and texturally pleasing to the user. Generally, ointments and *w*/*o* creams are less irritating. Creams contain emulsifiers and preservatives that may cause contact allergies [7]. Aqueous creams will not usually receive much satisfaction in the case of repellent due to their disappearing nature. Therefore, for the purpose of this research, an oil cream will be considered a suitable topical preparation.

## 2. Material and Methods

### 2.1. Milling of Seeds

The dried neem seed was milled using the local milling machine. A quantity of 1,020 g of neem seed was weighed and milled. After milling, 1,000 g of the powdered seed was stored in an airtight vessel.

### 2.2. Extraction and Evaluation of the Neem Seed Oil

By adopting the method of [9], hexane was used as the solvent for the extraction of the neem oil. Using a seed to solvent ratio of 1:10, the seed was allowed to soak in the solvent at room temperature for ten days. The solvent was then filtered through a filter paper to remove the coarse seed materials into preweighed sterile containers. The filtrate was then evaporated at 68°C to get rid of the hexane. The volume of the extracted oil was recorded and kept at room temperature.

#### 2.2.1. Determination of the Physicochemical Properties of the Oil

Physicochemical tests that include odor, color, saponification value, acid value, and moisture content were conducted to ensure that the oil obtained was of good quality and do not contain any traces of hexane.

#### 2.2.2. Determination of pH Levels

The pH of the oil was determined using the Oakton pH meter (PC 700), which was previously calibrated using standard solutions of known pH (4.0, 7.0, and 9.0). The determination was done for six weeks. The extracted oil with a volume of 20 mL was homogenized in a beaker. The electrode was removed from the storage solution and rinsed thoroughly with distilled water. The probe was gently wiped with tissue paper and dipped into the oil. The value obtained was recorded. This was done in triplicate, and an average was found. The electrodes were replaced after rinsing with distilled water.

#### 2.2.3. Determination of Acid Value of the Oil

The acid value of the oil was determined using the AOCS method [10]. Extracted neem seed oil weighing 5.0 g was placed in a dried conical flask. About 25 mL of absolute ethanol was added. Then 2-3 drops of phenolphthalein were also added.

It was later heated on a Fisher scientific water bath at 65°C for ten minutes, and then it was allowed to cool. The solution was titrated against 0.1 N KOH in a burette until a pink color appeared (end point). The volume of the KOH at the end point was recorded.

The acid value (AV) was calculated using the following formula:

(1) $$\begin{array}{c} \text{AV}=\frac{V\times N}{W}, \end{array}$$

where AV is the acid value, *V* is the volume at the end point, *N* is the number of KOH, and *W* is the weight of the sample.

#### 2.2.4. Determination of the Saponification Value of the Oil

The saponification value of the extracted oil was determined using the AOCS method [10]. Approximately 5 g of the oil was weighed into a 250 mL conical flask. Alcoholic potassium hydroxide solution (0.5 N) solution was prepared by dissolving 28 g of KOH in 1 liter of alcohol. A quantity (25 mL) of the alcoholic KOH solution was added to the conical flask containing the oil. A reflux condenser was attached to the flask, and the content was heated on a boiling water bath for 1 hour with occasional shaking.

While the solution was still hot, three drops of phenolphthalein indicator were added, and the excess potassium hydroxide was titrated against 0.5 N HCL (the volume of hydrochloric acid used at the end point represents *S*).

The procedure was repeated on a blank sample (without the oil) using 25 mL of KOH (the volume of hydrochloric acid used at the end point represents *B*).

This was calculated using the following formula:

(2) $$\begin{array}{c} \text{SP}=56.1\left(B-S\right)\times \frac{N}{M}, \end{array}$$

where SP is the saponification value, *B* is the volume of the blank solution (without oil), *S* is the volume of HCl (with oil), and *M* is the mass of the oil.

#### 2.2.5. Moisture Content Test of the Extracted Neem Oil

The moisture content of the oil was determined using the oven drying method (BC, 2004). A specific quantity of the oil (5 g) was weighed and transferred into a dried Petri dish, which was preweighed. The dish was placed in the hot air oven, which was thermostatically controlled at 105°C for 5 hours. The sample was removed and placed in a desiccator to cool at room temperature, and the weight was recorded later. It was then dried again for 30 minutes, cooled, and weighed again. The experiment was replicated until a constant weight was reached. The % moisture content was calculated as follows:

(3) $$\begin{array}{c} \%\text{ Moisture}=\frac{W_{i}-W_{f}}{W_{i}}\times 100, \end{array}$$

where *W*_*i*_ and *W*_*f*_ are the weight before and after drying, respectively.

### 2.3. Formulation Studies

#### 2.3.1. Preparation of Oil Cream Containing Neem Seed Oil as the Active Ingredient

Dried magnesium sulfate (0.5 g) and chlorocresol (0.1 g) were dissolved in enough warm water to produce a weight of 10 g. Wool alcohol ointment (55 g) was weighed and heated to 60°C. Sufficient fragrances were added to the heated ointment base with vigorous stirring using a homogenizer. Gradually, the aqueous mixture containing dried magnesium sulfate and chlorocresol was added followed by neem seed oil until a smooth uniform cream was obtained.

This mixture was then allowed to cool, stored in an appropriate container, and labelled. The same procedure was followed to prepare 4 other different concentrations: 12.5% *v*/*w*, 15% *v*/*w*, 17.5% *v*/*w*, and 20% *v*/*w* [11].

#### 2.3.2. Determination of Organoleptic Properties

These tests were conducted to identify the physical properties as well as the acceptance level of the creams on the market. An interview guide was distributed to 30 respondents after testing them with the formulated cream. The results obtained were analyzed using SPSS 16.0 version to process the data where individual respondents indicated the texture, odor, and color of the formulated neem seed oil cream.

#### 2.3.3. Determination of pH of Neem Seed Oil Cream

After the formulation of the cream at the various concentration levels, the pH was determined. Neem seed formulated cream of 20 mL was measured. The electrode of the pH meter was removed from the storage solution and rinsed thoroughly with distilled water. Furthermore, the probe was gently wiped with tissue paper and dipped into the cream. The pH value was recorded. The electrode was replaced after rinsing with distilled water. Triplicate measurements were made.

The pH was conducted at all concentration levels, that is, at 10% *v*/*w*, 12.5% *v*/*w*, 15% *v*/*w*, 17.5% *v*/*w*, and 20% *v*/*w*. Moreover, the tests were conducted on day 1 and after 30 days since it is an extemporaneous preparation.

#### 2.3.4. Testing for Viscosity of Neem Seed Oil Cream

The viscosity of the cream was determined using the Rapid Viscosity Analyzer (RVA). A mass of 10 g of the cream was weighed into the canister. The paddle was inserted and stirred up and down for the cream to homogenize. The paddle was then inserted into the RVA, and the instrument was run for 18 minutes at 50 rpm at 25°C. The results were read by the analyzer in centipoise. This was triplicated for all the other concentrations, and the average was found.

#### 2.3.5. Spreadability Studies on the Cream

The method described by [12] was modified and used in the determination as follows: a mass of 0.1 g of the cream was placed in a circle of 1 cm diameter premarked on a glass plate, over which a second glass was placed. A weight of 2 g was allowed to rest on the upper glass plate. The spreading time of the cream was measured. This was triplicated for all the concentrations, and the average was calculated.

The spreadability of the cream was calculated using the following formula:

(4) $$\begin{array}{c} S=\frac{L}{T}\times M, \end{array}$$

where *S* is the spreadability, *M* is the mass of the upper glass slide, *T* is the time in seconds, and *L* is the length of the glass slide.

#### 2.3.6. Determination of Microbial Loads

One percent (1%) of neem seed oil cream of 10% *v*/*w* concentration was prepared with sterile water, and 1 mL of the resultant mixture was inoculated into 20 mL of previously sterilized nutrient agar, MacConkey agar, Sabouraud agar, bismuth sulfite agar, mannitol salt agar, and cetrimide agar. The agars were then incubated at 37°C for 48 hours, and the growth of precise mechanisms depending on the selective media that was utilized was read as absent or present. The same procedure was followed for 12.5% *v*/*w*, 15% *v*/*w*, 17.5% *v*/*w*, and 20% *v*/*w* for freshly prepared cream and after 1 month, respectively [11].

#### 2.3.7. Skin Irritancy Studies on the Cream

Healthy female albino Wistar mice weighing between 28 and 30 g were obtained from the Department of Pharmacology, KNUST. Twenty-four hours before the experiment, furs from the backs of all the mice were clipped. Later, 0.5 mL of 10% *v*/*w* formulated neem seed oil cream was applied in a test site, while the untreated areas served as control [13]. This was done for all concentrations. The test site of application was examined critically at 1 hour and then at 24 hours, 48 hours, and 72 hours, respectively, to observe dermal reactions for all the concentrations.

### 2.4. In Vitro Characterization

#### 2.4.1. Anopheles Repellent Activity Studies on the Cream Using a Marketed Repellent Product as a Reference

The repellent experiments using human blood samples were approved by the Committee on Human Research Publication and Ethics (CHRPE), Kwame Nkrumah University of Science and Technology (KNUST), Ghana. The samples used in the repellency test are detailed in Table 1.

The repellent activity carried out by [14] was modified and used in the determination as follows:

The anopheles mosquitoes used for the experiment were laboratory-reared. In rearing the mosquitoes, a glass container was filled with water collected from a filthy environment. The container was covered with a clean white net and monitored under close observation. Eggs on the surface of the water began to wiggle after 48 hours. Full-grown mosquitoes began to hatch about 96 hours after collection. The hatched mosquitoes had no access to a blood meal. One liter of blood each in four different infusion bags was used for the experiment. The neem seed oil of 1 mL was applied to the first infusion bag containing blood labelled (A). Formulated neem seed oil cream starting with the lowest concentration to the highest was applied to the second infusion bag one after the other labelled B, C, D, E, and F, respectively.

A marketed mosquito repellent containing DEET (13%) labelled G was applied to the third infusion bag with blood. A fourth infusion bag containing blood without any cream was used as a control. Finally, an empty infusion bag was used as second control labelled. The first control bag without blood was hanged in the glass chamber, and the number of mosquito landings were recorded in a time interval of three minutes. The second infusion bag containing blood was hanged in the glass chamber, and the number of mosquito landings were recorded in the same time interval. The treatment infusion bag with blood smeared with 1 mL of neem seed oil was hanged in the same cage, and the number of mosquito landings were also recorded over a period of three minutes. The process was also repeated for another infusion bag with blood for the formulated neem seed oil cream starting with the lowest concentration. Finally, the same process was repeated for 1 mL of the marketed repellent product labelled *G*, and the number of mosquito landings were also recorded. Each infusion bag of blood was tested at various concentrations of the formulated neem seed oil cream using 1 mL of the cream, starting with the lowest concentration of 10% *v*/*w*, 12.5% *v*/*w*, 15% *v*/*w*, 17.5% *v*/*w*, and 20% *v*/*w*.

The entire experiment was conducted using the same species of mosquitoes at the same time interval (3 minutes). The mosquito repellent activity of the various formulations was compared with the reference standard statistically using GraphPad Prism 6 version.

## 3. Results and Discussions

Traditionally, the neem oil is used alone for years as a repelling agent that inferred the theory of getting a suitable and acceptable topical formulation for patients' use. Therefore, several physicochemical characteristics were determined on the oil (Table 2).

A pH is a physical unit used to measure the degree of acidity or alkalinity of a solution. Many important properties of a solution can be determined from an accurate measurement of pH, including the acidity of a solution and the extent of reaction in a solution. The pH of the neem seed oil was 5.56. This falls within the acceptable range of the pH of the skin, therefore making the oil highly suitable to be used for formulation without causing any skin irritation.

The acid value is a common parameter in the specification of fats and oils. It is defined as the number of mg of KOH required to neutralize the organic acids present in 1 g of fat or oil. It is also a measure of free fatty acids present in the oil. An increase in free fatty acids in the oil is an indication of hydrolysis of triglycerides. Such reaction occurs by the action of lipase enzyme, and this is an indicator of inadequate processing and storage conditions such as high temperature and relative humidity. Therefore, a lower acid value index of 9.32 mg/g is an indication of the good lubricating properties of the oil [5].

The saponification value is the number of milligrams of potassium hydroxide required to neutralize the free fatty acids resulting from the complete hydrolysis of 1 g of fat. The saponification value provides information on the average molecular weight of oil. The high saponification value of 204.67 g/mg showed a high proportion of saponifiable fats, which confirms the good quality of the oil for the production of soap [15].

The determination of moisture content is important in the pharmaceutical industry because it influences the stability of pharmaceutical products. The presence of moisture encourages microbial growth. Moisture content affects the physical as well as the chemical properties of a pharmaceutical product. The moisture value was 1.72 g, which was very low. This indicates a lesser propensity for microbial contamination of the oil [15].

The percentage yield for the extracted neem seed oil was 50.1% *w*/*w*. This means that the extraction process and the organic solvent used were suitable. The slight modification of the extraction process such as soaking it in the organic solvent for a longer period of days contributed to good yield.

### 3.1. Evaluation of Organoleptic Properties of the Formulated Neem Seed Oil Cream

These are tests that were conducted to identify the physical properties such as color, odor, and texture of the cream. The physical properties were determined in Table 3.

It is very necessary that the physical characteristics such as its texture, color, and odor of an intended formulation are properly examined to please the user (Table 3). For instance, the smell of the neem seed oil is pungent garlic and for that matter not very pleasant for direct application to the skin. Hence, a combination of fragrances was used to make the formulation appear very pleasing for patients' use.

### 3.2. pH Studies on Neem Seed Oil Cream at Varied Concentrations on Day 1 and Day 30

**Figure 1:**
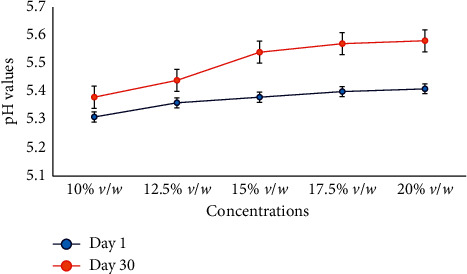
pH of different concentrations of neem seed oil cream on day 1 and day 30.

After 30 days, the cream formulation was tested for the pH according to their concentration levels, and the results are presented in Figure 1.

The pH of the formulation was determined in order to be certain that the formulation can be used without the risk of irritancy to the skin [16]. The changes in the pH of a formulation are a sign of chemical decomposition, most probably of a hydrolytic nature, and if detected are reason to return a product. The pH of the neem seed oil cream formulations with concentrations of 10% *v*/*w* to 20% *v*/*w* is graphically represented in Figure 1. According to [5], Azadirachtin, which is the major constituent of neem oil, is most stable in slightly acidic aqueous solutions between pH 4 and 6 at room temperature but unstable in mildly alkaline and strongly acidic solutions. Thus, it is observed to be favorable when pH for formulations generally falls around pH 5.5 and 6.5 showing no degradation of any kind. The pH was found to be between 5. 3 and 5.7 for the formulation, which was also close to the neutral pH. Hence, the formulation can be used without causing any skin irritation. This also showed that the selected constituents of the formulation did not alter the pH of the neem seed oil cream. Conclusively, this means that the oil cream formulation of the neem seed oil is user-friendly and could be applied by all.

### 3.3. Viscosity Studies on Neem Seed Oil Cream at Varied Concentrations on Day 1 and Day 30

**Figure 2:**
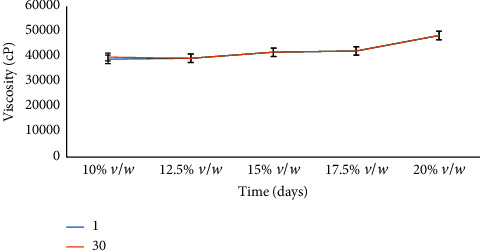
Viscosity studies of neem seed oil cream at their various concentrations.

In addition, the viscosity was equally tested on the cream after 30 days. This was performed on all the concentrations, and the results are shown in Figure 2.

Viscosity is a measure of a fluid's resistance to flow; it describes the internal friction of a moving liquid. There was no significant difference across the freshly prepared cream and the 30-day period of study for each of the concentrations of the formulations with respect to viscosity. The viscosities of the formulations of neem seed oil cream (10% *v*/*w*–20% *v*/*w*), which is graphically represented in Figure 2, means that the rate of concentration is directly proportional to the viscosity of the formulated cream. The higher the concentration, the greater the viscosity. An increase in viscosity across the concentration range is a result of an increase in the concentration of neem oil, which itself is a static oil.

**Figure 3:**
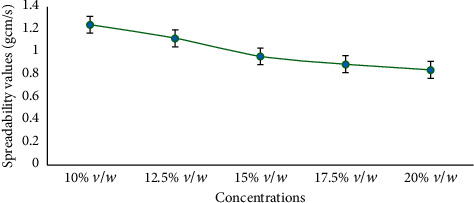
Spreadability studies of the neem seed oil cream at their various concentrations.

The spreadability of neem seed oil cream at varied concentrations are shown in Figure 3.

The spreadability of the formulation embraces the principle of the time or speed that it takes the formulation to spread. A formulated cream should spread easily without too much drag and should not produce greater friction in the rubbing process. As shown in Figure 3, it was realized that at a concentration of 10% *v*/*w*, the flow value was 1.25 gcm/s. When concentration increased to 12.5% *v*/*w*, the flow property reduced to 1.1 gcm/s and subsequently reduced to 0.8gcm/s at concentration 20% *v*/*w* [5]. Unlike viscosity, where concentration is directly proportional, the spreadability of the neem oil cream was inversely proportional to its concentration, that is, whereas the concentration increases, the spreadability rather declines.

### 3.4. Evaluation of Microbial Load of the Formulated Cream

The microbial quality for all constituents intended for any pharmaceutical formulation should be carefully assessed before they are used. As a result, several microbial quality tests were conducted to verify if they conform to the standard specifications and conditions (Table 4).

From the results in Table 4, it can be inferred that the formulation was free from pathogens that are known from clinical research to cause infections. Similarly, the total viable or aerobic count was within the acceptable criteria (<10^3^ cfu/g). Organisms such as *Staph aureus*, *Pseudomonas aeruginosa*, *Salmonella typhi*, and *Escherichia coli* are linked to microbial contamination. These microorganisms must be absent from the drug product [17]. However, appropriate storage conditions are advised to ensure prolonged shelf life.

### 3.5. Repellency Studies at Various Concentrations

**Figure 4:**
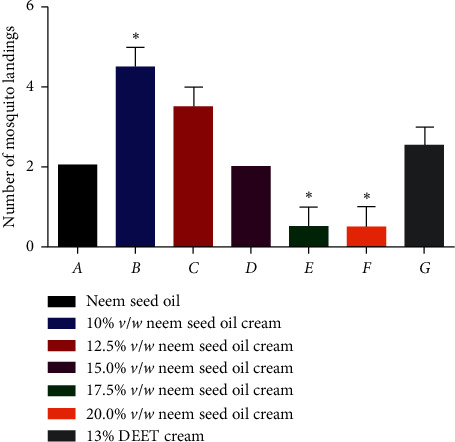
Number of mosquito landings on various concentrations of the neem seed oil cream after three minutes. ^*∗*^Values are significantly different *p* ≤ 0.05 as compared with the reference drug (DEET).

The repellent activities of the neem seed oil cream and reference artificial repellent cream were assessed using an in vitro study approach. The results indicated high repellency in the formulated cream as shown in Figure 4.

Reduction in mosquito landings ranged from 37.5% to 87.5%. The concentrations of the neem seed oil cream are directly proportional to its repellency. The lowest concentration (10% *v*/*w* repelled the least (37.5%), while the highest concentration (20% *v*/*w*) repelled the most (87.5%). Based on the statistical analysis obtained for the repellent activities of the various formulations (Figure 4), it can be deduced that there was a significant difference between 17.5% *v*/*w* and 20% *v*/*w* compared with the standard (*p* value ≤0.05) in terms of mosquito landings. The high level of significance shows that formulated neem seed oil cream of 17.5% *v*/*w* and 20% *v*/*w* is able to give a higher repellent effect compared with the standard. Therefore, these two concentrations of the formulated neem seed oil cream are best suited for repellent effects. However, there was also a significant difference between 10% *v*/*w* neem seed oil cream and that of the reference (*p* value ≤0.05). The DEET showed a reduced number of mosquito landings indicating a higher repellency effect compared with the lowest concentration 10% *v*/*w* of the formulated oil cream.

### 3.6. Skin Irritation Tests of the Neem Seed Oil Cream

The skin irritation test is used to evaluate the potential of a formulation to cause skin irritation when used by a consumer. Results obtained after the application of the formulated cream are discussed in Table 5.

The formulated dosage form was designed for topical application; hence, there was the need to check for its irritation on the skin. Results for the skin irritation test indicated in Table 5 showed that for 20% *v*/*w*, there were traces of erythema particularly after 24 hours, 48 hours, ad 72 hours of application. This means that although the formulation with a concentration of 20% *v*/*w* expresses good repellency, it produces skin irritancy.

## 4. Conclusions

The neem seed oil was successfully extracted with a percentage yield of 50.1% *w*/*w*. This was followed by formulation in the wool alcohol ointment. The formulated neem seed oil cream passed various pharmacopoeia tests; however, erythema was observed after 24 hours for 20% *v*/*w*.

## Figures and Tables

**Table 1:** List of samples for the repellency test.

Sample	Key
(1) neem seed oil	A
(2) 10.0% *v*/*w* neem seed oil cream	B
(3) 12.5% *v*/*w* neem seed oil cream	C
(4) 15.0% *v*/*w* neem seed oil cream	D
(5) 17.5% *v*/*w* neem seed oil cream	E
(6) 20.0% *v*/*w* neem seed oil cream	F
(7) 13% DEET cream (reference)	G

**Table 2:** Physicochemical characteristics of the neem oil.

Parameter	Values
pH	5.56 ± 0.12
Acid value (mg KOH/g)	9.32 ± 0.02
Saponification value (mg KOH/g)	204.67 ± 2.08
Moisture content	1.72 ± 0.015
Percentage yield	50.1% *w*/*w*
Color	Yellowish-brown
Odor	Pungent garlicky

**Table 3:** Organoleptic characteristics of formulated neem seed oil cream.

Characteristics of the formulated cream	
Color	Creamy brown
Odor	Pleasant
Texture	Smooth

**Table 4:** Microbial loads of the formulated cream.

Media						
Formulation	A	B	C	D	E	F
10% *v*/*w*	+	−	+	−	−	−
12.5% *v*/*w*	+	−	+	−	−	−
15% *v*/*w*	+	−	+	−	−	−
17.5% *v*/*w*	+	−	+	−	−	−
20% *v*/*w*	+	−	+	−	−	−

A = Nutrient agar, B = MacConkey agar, C = Sabouraud agar, D = Mannitol salt agar, E = Cetrimide agar, F = Bismuth sulfite agar, + = Growth present, and – = Growth absent.

**Table 5:** Skin irritation tests of neem seed oil cream.

Reaction	Time																			
	10% *v*/*w*				12.5% *v*/*w*				15% *v*/*w*				17.5% *v*/*w*				20% *v*/*w*			
	1 h	24 h	48 h	72 h	1 h	24 h	48 h	72 h	1 h	24 h	48 h	72 h	1 h	24 h	48 h	72 h	1 h	24 h	48 h	72 h
Irritation	–	–	–	–	–	–	–	–	–	–	–	–	–	–	–	–	–	+	+	+
Inflammation	–	–	–	–	–	–	–	–	–	–	–	–	–	–	–	–	–	–	–	–
Death	–	–	–	–	–	–	–	–	–	–	–	–	–	–	–	–	–	–	–	–

+ = Present and – = Absent.

## Data Availability

The data used to support the findings of this study are included in the article and also available from the corresponding author upon request.
